# Higher preconceptional maternal body mass index is associated with faster early preimplantation embryonic development: the Rotterdam periconception cohort

**DOI:** 10.1186/s12958-021-00822-0

**Published:** 2021-09-18

**Authors:** Linette van Duijn, Melek Rousian, Jeffrey Hoek, Sten P. Willemsen, Eva S. van Marion, Joop S. E. Laven, Esther B. Baart, Régine P. M. Steegers-Theunissen

**Affiliations:** 1 Department of Obstetrics and Gynecology, Erasmus MC, University Medical Center, Dr. Molewaterplein 40, Rotterdam, 3015 GD The Netherlands; 2 Department of Biostatistics, Erasmus MC, University Medical Center, Rotterdam, The Netherlands; 3 Division of Reproductive Endocrinology and Infertility, Department of Obstetrics and Gynecology, Erasmus MC, University Medical Center, Rotterdam, The Netherlands; 4 Department of Developmental Biology, Erasmus MC, University Medical Center, Rotterdam, The Netherlands

**Keywords:** Body mass index, Time-lapse, Morphokinetics, Embryo development, Preimplantation

## Abstract

**Background:**

Overweight and obesity affect millions of people globally, which has also serious implications for reproduction. For example, treatment outcomes after in vitro fertilisation (IVF) are worse in women with a high body mass index (BMI). However, the impact of maternal BMI on embryo quality is inconclusive. Our main aim is to study associations between preconceptional maternal BMI and morphokinetic parameters of preimplantation embryos and predicted implantation potential. In addition, associations with clinical IVF outcomes are investigated.

**Methods:**

From a tertiary hospital, 268 women undergoing IVF or IVF with intracytoplasmic sperm injection (ICSI) were included; 143 normal weight, 79 overweight and 46 obese women. The embryos of these women were cultured in the EmbryoScope, a time-lapse incubator. The morphokinetic parameters of preimplantation embryos and predicted implantation potential, assessed by the KIDScore algorithm were longitudinally evaluated as primary and secondary outcomes, respectively. The tertiary outcomes included clinical outcomes, i.e., fertilization, implantation and live birth rate.

**Results:**

After adjustment for patient- and treatment-related factors, we demonstrated in 938 embryos that maternal BMI is negatively associated with the moment of pronuclear appearance (β_tPNa_ -0.070 h (95%CI -0.139, -0.001), *p* = 0.048), pronuclear fading (β_tPNf_ -0.091 h (95%CI -0.180, -0.003), *p* = 0.043 and the first cell cleavage (β_t2_ -0.111 h (95%CI -0.205, -0.016), *p* = 0.022). Maternal BMI was not significantly associated with the KIDScore and tertiary clinical treatment outcomes. In embryos from couples with female or combined factor subfertility, the impact of maternal BMI was even larger (β_tPNf_ -0.170 h (95%CI -0.293, -0.047), *p* = 0.007; β_t2_ -0.199 h (95%CI -0.330, -0.067), *p* = 0.003). Additionally, a detrimental impact of BMI per point increase was observed on the KIDScore (β -0.073 (se 0.028), *p* = 0.010).

**Conclusions:**

Higher maternal BMI is associated with faster early preimplantation development. In couples with female or combined factor subfertility, a higher BMI is associated with a lower implantation potential as predicted by the KIDScore. Likely due to power issues, we did not observe an impact on clinical treatment outcomes. However, an effect of faster preimplantation development on post-implantation development is conceivable, especially since the impact of maternal BMI on pregnancy outcomes has been widely demonstrated.

**Supplementary Information:**

The online version contains supplementary material available at 10.1186/s12958-021-00822-0.

## Introduction

Overweight and obesity affect millions of people of all ages, genders, ethnicities and income levels [1]. Although the pathophysiology of adiposity is highly complex and multifactorial, it is fundamentally caused by a positive energy imbalance and influenced by genetic and numerous environmental factors [2, 3]. Surplus energy is stored as fat, which leads to a disruption of numerous physiological processes on endocrine, immune and vascular levels [4]. This explains why an elevated body mass index (BMI) is associated with various non-communicable diseases, such as diabetes type 2 and cancer [5, 6].

Obesity and overweight not only increase the risk of non-communicable diseases, but also can impact reproduction [7]. As almost half of the women in the reproductive period are overweight or obese, this has serious consequences. Adiposity affects metabolic and endocrine processes involved in fertility, which leads to an increased risk of miscarriages, reduced conception rate and anovulation [8–10]. Therefore, overweight and obesity are likely overrepresented in women receiving fertility treatment, such as in vitro fertilization (IVF) [11].

Outcomes after IVF treatment are poorer in women with a high BMI compared to normal weight women [12–14]. The mechanisms by which adiposity affects reproduction are not yet fully understood. It is suggested that obesity interferes with biological processes and pathways at endocrine, follicular, uterine and embryonic levels [15–18]. For example, obesity increases follicular fluid concentrations of lipids, metabolites and inflammatory markers, and impacts gene expression of cumulus cells, which can impair oocyte development [19, 20]. Interestingly, the impact of maternal BMI on embryo quality is inconclusive [18, 21–23].

Since three decades, preimplantation embryo development can be closely observed with time-lapse imaging [24]. This technique is increasingly used to study associations between embryo development and implantation, and to improve embryo selection by algorithms such as the KIDScore [25]. Prospective randomized trials report conflicting results on the improvement of success rates after embryo selection based on time-lapse parameters, and also indicate that these parameters are subject to patient-related factors [26, 27]. Moreover, previous studies investigating the impact of BMI on these parameters report conflicting results and are exclusively performed in cycles with intracytoplasmic sperm injection (ICSI) [28, 29].

From this background, the main aim of this study is to investigate the hypothesis that a high maternal BMI is detrimentally associated with preimplantation embryo quality, as assessed by developmental time-lapse parameters and predicted implantation potential (KIDScore). In addition, we also investigated associations with clinical treatment outcomes after IVF/ICSI treatment.

## Methods

### Study design

The data used for this study was collected between May 2017 and December 2019 as part of the Virtual Embryoscope study. This is an ongoing prospective sub-study of the Rotterdam Periconception Cohort, an observational open prospective tertiary hospital-based cohort, embedded in the outpatient clinic of the Department of Obstetrics and Gynaecology of the Erasmus MC, University Medical Center, Rotterdam, The Netherlands [30]. The Rotterdam Periconception Cohort focuses on periconceptional influences on reproductive success and adverse pregnancy outcomes and health of the offspring up to 1 year of age. For this study, subfertile couples with an indication for IVF/ICSI treatment, aged 18 years or older with adequate understanding of the Dutch language were eligible for participation.

**Fig. 1:**
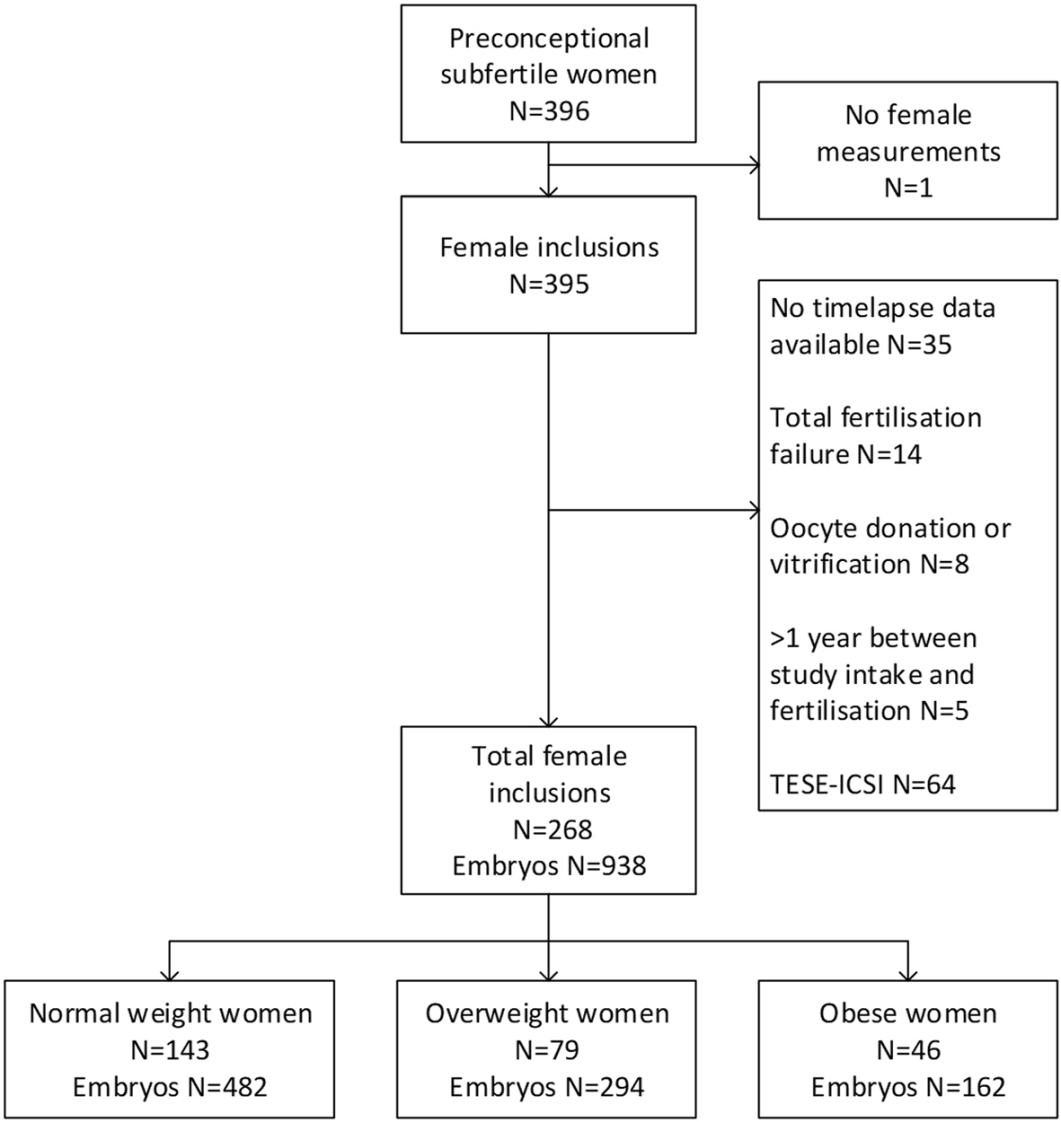
Flowchart of the VIRTUAL EmbryoScope study population embedded in the Rotterdam Periconception Cohort. Abbreviations: TESE, testicular sperm extraction. ICSI, intracytoplasmic sperm injection

For this study, couples were excluded if: 1) no fertilization occurred and no embryos were available (*n* = 14); 2) the embryos were not cultured in the EmbryoScope (*n* = 35); 3) IVF/ICSI treatment was performed with donated or vitrified oocytes (*n* = 8); 4) treatment was performed after > 1 year following inclusion in the study (*n* = 5) or 5) ICSI was performed with testicular extracted sperm (Fig. 1).

### In vitro fertilization procedures and time-lapse imaging

Ovarian stimulation, oocyte retrieval and IVF/ICSI procedures were performed as previously described [31, 32]. Women underwent ovarian stimulation with either recombinant follicle stimulating hormone (rFSH) or urinary FSH, with gonadotrophin-releasing hormone (GnRH) agonist or GnRH-antagonist co-treatment. Ovarian stimulation protocols were standardized; the distribution of GnRH-agonist or -antagonist protocol reflects policy changes over time and not patient selection. FSH dosage was based on maternal age, BMI, antral follicle count, anti-müllarian hormone (AMH) level and prior response to gonadotrophins (if applicable). Final follicular maturation was triggered with human chorionic gonadotrophin (hCG) or a GnRH-agonist. Oocytes were collected 35 h later and cultured in SAGE human tubal fluid medium (HTF, CooperSurgical, Trumbull, CT, USA); supplemented with 5% human serum albumin (CooperSurgical) under an oil overlay (CooperSurgical).

After insemination, IVF oocytes were cultured overnight in drops of 100 µl HTF medium in universal GPS dishes (CooperSurgical) under oil. The next morning, only fertilized dipronucleate oocytes were transferred to an EmbryoSlide (Vitrolife, Goteborg, Sweden) for culture in the EmbryoScope™ time-lapse incubator (Vitrolife). ICSI oocytes were denuded and transferred to an EmbryoSlide directly after sperm injection. Injected oocytes or embryos were cultured individually in 25 µl of SAGE 1-Step medium (Cooper Surgical) under 1.4 ml oil. Culture in the EmbryoScope™ was conform conventional culture, performed using customized settings, with a temperature setting of 36.8 °C and in an atmosphere containing 7% O_2_ and 4.5% CO_2_. This atmosphere was validated to result in a pH of the SAGE 1-Step culture medium of 7.2–7.3.

Images were automatically recorded every 10 min after exposure to a single red LED (635 nm, < 0.1 s per image) with a monochrome CCD camera. On day 3 after fertilization, embryo evaluation and selection for embryo transfer (ET) was based on conventional morphology, i.e. number of blastomeres, fragmentation, size equality, and signs of early compaction, without the support of time-lapse information. Selection of embryos for cryopreservation was performed on day 4, based on the degree of compaction and fragmentation.

Two weeks after ET, implantation was biochemically confirmed by a positive β-hCG test. Pregnancy was confirmed by ultrasound at 7 and 12 weeks of gestation by the presence of a gestational sac and fetal heartbeat.

### Study parameters

Participants completed a preconceptional questionnaire covering demographic and lifestyle details. A researcher verified all data at study entry and measured anthropometrics. Subfertility diagnoses were retrieved from medical records and, when applicable, stratified according to the WHO classification of anovulation [33].

Time-lapse parameters were annotated manually according to the definitions of the ESHRE consensus for dynamic monitoring of human preimplantation development [34]. All freshly transferred and cryopreserved embryos were annotated for the following morphokinetic parameters: pronuclear appearance (tPNa), pronuclear fading (tPNf), t2, t3, t4, t5, t6, t7 and t8. tPNa was defined as the first frame in which both pronuclei had appeared, and tPNf as the first frame in which both had faded. Timing of reaching the 2-, 3-, 4-, 5-, 6-, 7-, and 8-cell stage was defined as t2, t3, t4, t5, t6, t7 and t8, respectively. Some of these parameters were used to assign each embryo a KIDScore (Supplemental table 1). This is a generally applicable embryo deselection tool based on 6 parameters, of which the lowest score (= 1) corresponds with a chance of implantation of 5%, whereas the highest score (= 5) corresponds with a 36% chance of implantation [25]. Validation of the KIDScore in our clinic showed that KIDScore 1 embryos implant in 23% of cases, increasing to 52% for KIDScore 5 embryos after SET. Internal validation of inter-observer reproducibility demonstrated extremely close agreement for the timings of tPNf until t5 (intraclass correlation coefficient (ICC) > 0.95). A moderate agreement was found for t6, t7 and t8 (ICC 0.23–0.40).

Clinical treatment outcomes were retrieved from medical records. Pre-transfer clinical treatment outcomes included: fertilization rate, which was calculated by dividing the number of fertilized oocytes by the number of metaphase II oocytes retrieved and embryo usage rate, which was calculated by dividing the number of usable embryos, i.e. all freshly transferred and cryopreserved, by the number of fertilized oocytes. Post-transfer clinical treatment outcomes included cumulative pregnancy rate, which was defined as an ongoing pregnancy resulting from either fresh ET or frozen-thawed ET from the studied treatment cycle within a 2 year follow-up period.

### Statistical analyses

Based on initial reports in human embryos, preimplantation embryos from obese women are expected to be developmentally delayed [35]. Based on culture of the first 900 embryos in our EmbryoScope™, average time needed from pronuclear disappearance to the 4-cell stage was 16.7 h (SD ± 5). The intra-cluster correlation coefficient (ICC) was 0.16. To show a delay of at least 2 h (0.4SD) in reaching the 4-cell stage, while correcting for statistical clustering of embryos derived from the same patient (1 + ([average no. of embryos/ patient]-1)*ICC = 1 + 4*0.1591 = 1.64), we needed 100* 1.64 = 164 embryos in each group to achieve an 80% power to detect this 2 h difference at α = 0.05.

Continuous baseline data were compared between women with normal weight, overweight and obesity, using Kruskall-Wallis tests for continuous data and chi-square tests for categorical data.

Analyses of morphokinetic parameters were performed on transferred and cryopreserved embryos. Since couples often have multiple embryos and embryos from a couple are likely to exhibit comparable developmental patterns, linear regression analyses are not appropriate. Therefore, we applied linear mixed models with time-lapse parameters as response variables and BMI as independent variable.

Proportional odds models were used to study the association between maternal BMI and the KIDScore, using the ordinal package in R (Rune Haubo B Christensen). This model is for ordinal outcomes like the KIDScore, with patient-specific intercepts to account for the correlation between sibling embryos.

Associations between BMI and continuous treatment outcomes, such as fertilization rate, were analyzed using linear regression. For associations between BMI and dichotomous outcomes, such as a positive β-hCG-test, logistic regression was applied.

All associations were studied with maternal BMI as a continuous variable. Two models were constructed for analyses on morphokinetic parameters and the KIDScore; a crude model without adjustments and the adjusted model with adjustments for maternal age, fertilization method, type of ovarian stimulation and paternal BMI and age. Analyses on treatment outcomes were adjusted for maternal age and type of ovarian stimulation. Post-hoc analyses were performed with maternal BMI divided into categories of normal weight (BMI ≤ 25 kg/m^2^), overweight (BMI > 25 kg/m^2^) and obese (BMI > 30 kg/m^2^), with normal weight as reference category. Furthermore, we stratified analyses of morphokinetic parameters for fertilisation method, as a 2-h delay between insemination and fertilisation has been suggested for IVF embryos [36]. In addition, we performed sub-analyses of embryos of couples with only a female subfertility diagnosis (e.g. endometriosis or PCOS) and a male partner with normal semen parameters, or combined female and male factor subfertility. All analyses were performed in SPSS statistics 25.0 (IBM, Armonk, USA) and R (R: A language and Environment for Statistical Computing, version 3.1.3, 2015 for Windows, R Core Team, Vienna, Austria). Two-sided *p*-values < 0.05 were considered significant.

## Results

### Baseline characteristics

A total of 268 women (*n* = 938 embryos) were included, of whom 143 were of normal weight (*n* = 482 embryos), 79 overweight (*n* = 294 embryos) and 46 obese (*n* = 162 embryos) (Fig. 1). Baseline characteristics were comparable between the three groups, except that normal weight women were more often highly educated than overweight and obese women (62.7, 51.9 and 30.4%, respectively, *p* = 0.004) (Table 1). Additionally, types of female type subfertility are not significantly different between the three groups (Supplemental table 2).

**Table 1:** Baseline characteristics of the VIRTUAL EmbryoScope study population (*n* = 268)

	Normal weight women		Overweight women		Obese women		*P*-value	Missing
	*N* = 143		*N* = 79		*N* = 43			
	Median/N	IQR/%	Median/N	IQR/%	Median/N	IQR/%		
Maternal factors								
Age, years	34.3	30.5–38.3	32.9	29.2–36.6	36.3	30.3–39.6	0.110	0
Geographic origin, Western	119	83.3	68	86.1	37	80.4	0.709	1
Eductional level							**0.004**	1
Low	6	4.2	6	7.6	5	10.9		
Intermediate	47	33.1	27	58.7	32	40.5		
High	89	62.7	41	51.9	14	30.4		
Folic acid supplements, yes	135	95.1	75	94.9	42	91.3	0.632	1
Vitamins, yes	80	60.6	37	50.7	17	41.5	0.073	22
Alcohol, yes	63	44.4	27	34.2	18	39.1	0.328	1
Cigarettes, yes	19	13.4	12	15.2	7	15.2	0.914	1
Treatment factors								
Cause of subfertility							0.987	0
Female factor	44	30.8	22	27.8	12	26.1		
Male factor	51	35.7	30	38.0	17	37.0		
Combined	28	19.6	17	21.5	9	19.6		
Unexplained	20	14.0	10	12.7	8	17.4		
ICSI, yes	79	55.2	50	63.3	28	60.9	0.478	0
Oocytes aspirated	8	5–12	9	6–14	8	5–12	0.278	2
Ovarian stimulation, GnRH-agonist	26	18.2	18	22.8	12	26.1	0.459	0

Significant differences are depicted in bold

*IQR* Interquartile range, *ICSI* Intracytoplasmic sperm injection

### Morphokinetic parameters

Linear mixed model analyses showed negative betas for all morphokinetic parameters, indicating a faster development for every increase in BMI point (Table 2). However, this was only significant for tPNf (β_crude_ -0.119 h (95%CI -0.206, -0.031), *p* = 0.008; β_adjusted_ -0.091 h (95%CI -0.180, -0.003), *p* = 0.043) and t2 (β_crude_ -0.142 h (95%CI -0.235, -0.049), *p* = 0.003; β_adjusted_ -0.111 h (95%CI -0.205, -0.016), *p* = 0.022). Stratification for fertilization method demonstrated that the negative beta for tPNa is almost exclusively based on ICSI embryos (Table 3). Interestingly, sub-analyses of embryos from couples with female or combined factor subfertility showed an even larger impact of BMI on tPNf (β_crude_ -0.164 h (95%CI -0.286, -0.042), *p* = 0.009; β_adjusted_ -0.170 h (95%CI -0.293, -0.047), *p* = 0.007) and t2 (β_crude_ -0.194 h (95%CI -0.323, -0.064), *p* = 0.004; β_adjusted_ -0.199 h (95%CI -0.330, -0.067), *p* = 0.003) (Table 4).

**Table 2:** The impact of maternal BMI on morphokinetic parameters

Morphokinetic parameter	Crude		Adjusted		Missing
	Beta (95%CI) hours	*p*-value	Beta (95%CI) hours	*p*-value	
tPNa	-0.074 (-0.163, 0.015)	0.102	-0.070 (-0.139, -0.001)	**0.048**	448^a^
tPNf	-0.119 (-0.206, -0.031)	**0.008**	-0.091 (-0.180, -0.003)	**0.043**	23
t2	-0.142 (-0.235, -0.049)	**0.003**	-0.111 (-0.205, -0.016)	**0.022**	3
t3	-0.100 (-0.223, 0.023)	0.109	-0.039 (-0.168, 0.089)	0.548	5
t4	-0.122 (-0.246, 0.001)	0.053	-0.087 (-0.220, 0.047)	0.201	8
t5	-0.102 (-0.266, 0.061)	0.220	-0.053 (-0.229, 0.122)	0.549	13
t6	-0.100 (-0.263, 0.063)	0.229	-0.073 (-0.251, 0.105)	0.418	33
t7	-0.069 (-0.242. 0.103)	0.429	-0.014 (-0.204, 0.175)	0.881	65
t8	-0.002 (-0.178, 0.174)	0.982	0.067 (-0.125, 0.259)	0.492	151

Adjusted for maternal age, fertilization method, type of ovarian stimulation and paternal BMI and age

Significant differences are depicted in bold

*CI* Confidence interval

^a^In cases of regular IVF, embryos are only transferred to the EmbryoScope after PN inspection, thus tPNa cannot be observed

**Table 3:** The impact of maternal BMI on morphokinetic parameters, stratified for fertilisation method

Morphokinetic parameters	IVF *n* = 111 women				ICSI *n* = 157 women			
	Crude		Adjusted		Crude		Adjusted	
	Beta (95%CI) hours	*p*-value	Beta (95%CI) hours	*p*-value	Beta (95%CI) hours	*p*-value	Beta (95%CI) hours	*p*-value
tPNa^a^	0.429 (-1.647, 2.508)	0.232	n/a		-0.070 (-0.131, -0.010)	**0.024**	-0.071 (-0.140, -0.001)	**0.046**
tPNf	-0.103 (-0.247, 0.041)	0.158	-0.115 (-0.270, 0.040)	0.143	-0.091 (-0.187, 0.005)	0.064	-0.080 (-0.186, 0.026)	0.136
t2	-0.152 (-0.306, 0.002)	0.053	-0.146 (-0.311, 0.019)	0.083	-0.095 (-0.196, 0.007)	0.067	-0.146 (-0.311, 0.019)	0.083
t3	-0.085 (-0.290, 0.119)	0.409	-0.046 (-0.273, 0.180)	0.685	-0.071 (-0.214, 0.073)	0.333	-0.031 (-0.183, 0.121)	0.690
t4	-0.101 (-0.312, 0.109)	0.342	-0.086 (-0.315, 0.143)	0.459	-0.105 (-0.251, 0.042)	0.161	0.081 (-0.242, 0.080)	0.320
t5	-0.081 (-0.354, 0.192)	0.557	-0.036 (-0.337, 0.265)	0.812	-0.075 (-0.273, 0.123)	0.456	-0.052 (-0.265, 0.161)	0.630
t6	-0.024 (-0.293, 0.245)	0.858	-0.018 (-0.319, 0.283)	0.906	-0.120 (-0.323, 0.085)	0.251	-0.103 (-0.326, 0.120)	0.362
t7	-0.080 (-0.363, 0.203)	0.577	-0.027 (-0.348, 0.295)	0.870	-0.022 (-0.239, 0.193)	0.836	-0.002 (-0.239, 0.235)	0.984
t8	-0.078 (-0.373, 0.217)	0.601	-0.038 (-0.371, 0.294)	0.820	0.084 (-0.132, 0.301)	0.443	0.122 (-0.116, 0.360)	0.310

Adjusted for maternal age, type of ovarian stimulation and paternal BMI and age

Significant differences are depicted in bold

*CI* Confidence interval

^a^*n* = 5 for tPNa in IVF-population

**Table 4:** The impact of maternal BMI on morphokinetic parameters of embryos of couples with either a female factor or combined factor subfertility diagnosis (*n* = 476)

Morphokinetic parameter	Crude		Adjusted		Missing
	Beta (95%CI) hours	*p*-value	Beta (95%CI) hours	*p*-value	
tPNa	-0.300 (-0.256, 0.196)	0.790	-0.011 (-0.120, 0.098)	0.841	290
tPNf	-0.164 (-0.286, -0.042)	**0.009**	-0.170 (-0.293, -0.047)	**0.007**	0
t2	-0.194 (-0.323, -0.064)	**0.004**	-0.199 (-0.330, -0.067)	**0.003**	0
t3	-0.169 (-0.353, 0.015)	0.072	-0.097 (-0.287, 0.092)	0.311	0
t4	-0.214 (-0.397, -0.031)	0.022	-0.203 (-0.399, -0.008)	**0.042**	0
t5	-0.127 (-0.358, 0.105)	0.280	-0.060 (-0.308, 0.187)	0.629	3
t6	-0.138 (-0.371, 0.095)	0.243	-0.148 (-0.401, 0.104)	0.247	10
t7	-0.148 (-0.394, 0.099)	0.237	-0.097 (-0.366, 0.173)	0.478	27
t8	-0.096 (-0.358, 0.167)	0.472	-0.021 (-0.316, 0.273)	0.887	68

Adjusted for maternal age, fertilization method, type of ovarian stimulation and paternal BMI and age

Significant differences are depicted in bold

*CI* Confidence interval

Post-hoc analyses demonstrated a significantly positive beta for t8 in embryos of overweight women, when compared to embryos of normal weight women, (β_crude_ 1.744 h (95%CI 0.087, 3.401), *p* = 0.039; β_adjusted_ 2.541 h (95%CI 0.774, 4.308), *p* = 0.005) (Table 5). Embryos of obese women reached tPNf and t2 faster than embryos of normal weight women (tPNf: β_crude_ -1.065 h (95%CI -2.082, -0.047), *p* = 0.040; t2: β_crude_ -1.311 h (95%CI -2.934, -0.227), *p* = 0.018; t2: β_adjusted_ -1.101 h (95%CI -2.195, -0.008), *p* = 0.048).

**Table 5:** Differences in morphokinetic parameters of embryos from overweight and obese women, compared to embryos of normal weight women

Morphokinetic parameter	Crude				Adjusted				Missing
	Overweight		Obese		Overweight		Obese		
	Beta (95%CI) hours	*p*-value	Beta (95%CI) hours	*p*-value	Beta (95%CI) hours	*p*-value	Beta (95%CI) hours	*p*-value	
tPNa	-0.593 (-1.438, 0.252)	0.167	-0.404 (-1.422, 0.614)	0.433	-0.316 (-0.981, 0.349)	0.348	-0.628 (-1.401, 0.144)	0.110	448
tPNf	-0.737 (-1.588, 0.114)	0.089	-1.065 (-2.082, -0.047)	**0.040**	-0.127 (-0.984, 0.730)	0.770	-0.914 (-1.940, 0.111)	0.080	23
t2	-0.630 (-1.533, 0.273)	0.171	-1.311 (-2.394, -0.227)	**0.018**	-0.050 (-0.955, 0.856)	0.914	-1.101 (-2.195, -0.008)	**0.048**	3
t3	-0.395 (-1.583, 0.794)	0.514	-0.852 (-2.278, 0.573)	0.240	0.352 (-0.876, 1.580)	0.573	-0.228 (-1.711, 1.254)	0.762	5
t4	-0.768 (-1.969, 0.434)	0.210	-0.825 (-2.267, 0.617)	0.261	-0.239 (-1.523, 1.044)	0.713	-0.418 (-1.966, 1.131)	0.596	8
t5	0.571 (-0.989, 2.131)	0.472	-0.626 (-3.501, 0.249)	0.089	1.331 (-0.319, 2.982)	0.113	-1.080 (-3.079, 0.918)	0.288	13
t6	0.510 (-1.039, 2.059)	0.517	-1.644 (-3.526, 0.237)	0.086	1.191 (-0.470, 2.852)	0.159	-1.446 (-3.479, 0.587)	0.162	33
t7	0.942 (-0.683, 2.566)	0.542	-0.378 (-3.353, 0.598)	0.171	1.672 (-2.996, 1.264)	0.060	-0.866 (-2.996, 1.264)	0.424	65
t8	1.744 (0.087, 3.401)	**0.039**	-0.994 (-2.977, 0.989)	0.324	2.541 (0.774, 4.308)	**0.005**	-0.235 (-2.367, 1.896)	0.828	151

Adjusted for maternal age, fertilization method, type of ovarian stimulation and paternal BMI and age

Significant differences are depicted in bold

*CI* Confidence interval

### Implantation potential, predicted by the KIDScore

The association between maternal BMI and predicted implantation potential, assessed by the KIDScore, was studied by a proportional odds model. The crude model showed a non-significant effect estimate of -0.019 (se 0.015, *p* = 0.206), indicating a lower KIDScore for a higher BMI. The adjusted model demonstrated a comparable estimate (β -0.020 (se 0.017), *p* = 0.218).

Interestingly, sub-analyses of embryos from couples with either a female or a combined factor subfertility diagnosis demonstrated a significant impact of BMI on the KIDScore (β_crude_ -0.049 (se 0.025), *p* = 0.052; β_adjusted_ -0.073 (se 0.028), *p* = 0.010). These observations indicate that a higher maternal BMI has a detrimental impact on the predicted implantation potential of embryos of women with an underlying cause for their subfertility.

Post-hoc analyses of maternal BMI into categories also demonstrated non-significant associations between either overweight or obesity and predicted implantation potential (β_overweight_ 0.008 (se 0.014, *p* = 0.547); β_obesity_ -0.253 (se 0.174, *p* = 0.178)). Similar results were found in the adjusted model (β_overweight_ 0.126 (se 0.192, *p* = 0.403); β_obesity_ -0.260 (se 0.192, *p* = 0.177)).

### Pre- and post-transfer clinical treatment outcomes

Crude linear regression analysis was applied to investigate associations between maternal BMI and the tertiary outcomes. The association between maternal BMI and total number of fertilized oocytes showed that for every point increase in BMI, the total number of fertilized oocytes per patient increased 0.024 (95%CI -0.075, 0.124, *p* = 0.630), yet this was not significant (Table 6). Maternal BMI was also not significantly associated with other pre- or post-transfer clinical treatment outcomes, such as implantation rate (odds ratio 0.994 (95%CI 0.936, 1.054), *p* = 0.994).

**Table 6:** The impact of maternal BMI on IVF/ICSI treatment outcome parameters

	Crude		Adjusted	
Pre-transfer	Beta (95%CI)	*p*-value	Beta (95%CI)	*p*-value
Total fertilized oocytes	0.024 (-0.075, 0.124)	0.630	0.031 (-0.067, 0.129)	0.532
Fertilization rate	-0.003 (-0.009, 0.003)	0.329	-0.003 (-0.009, 0.003)	0.303
Total usable embryos	0.018 (-0.049, 0.085)	0.605	0.021 (-0.046, 0.088)	0.536
Usage rate	0.000 (-0.007, 0.007)	0.913	0.000 (-0.007, 0.007)	0.928
Post-transfer	OR (95%CI)	*p*-value	OR (95%CI)	*p*-value
Positive β-hCG-test *n* = 106	0.994 (0.936, 1.054)	0.994	0.997 (0.938, 1.060)	0.930
Gestational sac *n* = 97	0.997 (0.939, 1.059)	0.923	1.000 (0.940, 1.064)	0.998
Fetal heartbeat *n* = 90	0.985 (0.927, 1.047)	0.630	0.986 (0.926, 1.050)	0.663
Live born^a^ * n* = 61	0.998 (0.92, 1.069)	0.949	1.000 (0.932, 1.073)	0.992
Cumulative pregnancy^b^ * n* = 132	1.036 (0.977, 1.097)	0.238	1.044 (0.983, 1.109)	0.163

Adjusted for maternal age and type of ovarian stimulation

*CI* Confidence interval, *OR* Odds ratio

^a^missing *n* = 22 ^b^missing *n* = 8

Post-hoc analyses of pre- and post-transfer clinical treatment outcomes of overweight and obese women as separate groups demonstrated no significant associations, when compared to normal weight women (Tables 7 and 8).

**Table 7:** Post-transfer treatment outcome parameters per BMI category

	Normal weight women *n* = 143		Overweight women *n* = 79		Obese women *n* = 46		*P*-value
	N	%	N	%	N	%	
Positive β-hCG-test	70	43.8%	43	49.4%	20	35.1%	0.237
Gestational sac	62	38.8%	41	47.1%	18	31.6%	0.163
Fetal heartbeat	58	36.3%	36	41.4%	17	29.8%	0.369
Live born^a^	36	25.2%	28	34.1%	12	23.1%	0.257

^a^missing *n* = 22

**Table 8:** Differences in treatment outcome parameters for overweight and obese women, compared to normal weight women

	Crude				Adjusted			
	Overweight		Obese		Overweight		Obese	
Pre-transfer	Beta (95%CI)	*p*-value	Beta (95%CI)	*p*-value	Beta (95%CI)	*p*-value	Beta (95%CI)	*p*-value
Total fertilized oocytes	0.541 (-0.420, 1.501)	0.269	0.183 (-0.969, 1.335)	0.755	0.428 (-0.524, 1.379)	0.377	0.315 (-0.824, 1.455)	0.586
Fertilization rate	-0.019 (-0.079, 0.041)	0.542	-0.025 (-0.097, 0.047)	0.491	-0.015 (-0.076, 0.045)	0.617	-0.029 (-0.101, 0.044)	0.434
Total usable embryos	0.351 (-0.298, 1.000)	0.288	0.097 (-0.681, 0.876)	0.805	0.292 (-0.357, 0.940)	0.377	0.164 (-0.612, 0.941)	0.677
Usage rate	-0.029 (-0.097, 0.038)	0.394	0.012 (-0.070, 0.093)	0.777	-0.026 (-0.095, 0.042)	0.446	0.010 (-0.072, 0.092)	0.815
Post-transfer	OR (95%CI)	*p*-value	OR (95%CI)	*p*-value	OR (95%CI)	*p*-value	OR (95%CI)	*p*-value
Positive β-hCG-test	1.349 (0.750, 2.428)	0.318	0.843 (0.420, 1.693)	0.632	1.296 (0.705, 2.380)	0.404	0.895 (0.435, 1.842)	0.763
Gestational sac	1.459 (0.807, 2.640)	0.211	0.931 (0.459, 1.888)	0.843	1.392 (0.754, 2.752)	0.291	0.986 (0.473, 2.053)	0.970
Fetal heartbeat	1.191 (0.654, 2.170)	0.567	0.872 (0.426, 1.785)	0.709	1.114 (0.600, 2.068)	0.733	0.908 (0.433, 1.902)	0.798
Live born^a^	1.303 (0.665, 2.552)	0.440	1.051 (0.469, 2.354)	0.904	1.254 (0.632, 2.491)	0.517	1.125 (0.494, 2.560)	0.780
Cumulative pregnancy^b^	1.446 (0.823, 2.540)	0.199	1.229 (0.663, 2.545)	0.446	1.304 (0.723, 2.350)	0.378	1.510 (0.742, 3.074)	0.256

Adjusted for maternal age and type of ovarian stimulation

*CI* Confidence interval, *OR* Odds ratio

^a^missing *n* = 22 ^b^missing *n* = 8

## Discussion

### Summary of findings

This study aimed to investigate the hypothesis that an elevated BMI in women undergoing IVF/ICSI treatment has a detrimental impact on 1) preimplantation morphokinetic parameters until day 3 of development, 2) predicted implantation potential and 3) pre- and post-transfer clinical treatment outcomes. We observed that a higher maternal BMI is associated with a faster progression through the cleavage stages. No significant association of maternal BMI with predicted implantation potential, as assessed by the KIDScore algorithm, was shown. However, in embryos of couples with female or combined factor subfertility, maternal BMI was associated with faster early embryonic development and lower predicted implantation potential. In addition, no significant associations were shown between maternal BMI and the tertiary clinical treatment outcomes.

When maternal BMI was divided into categories, we observed delayed reaching of the 8-cell stage in embryos of overweight women, whereas embryos of obese women reach the 2-cell stage faster than embryos of normal weight women. Morphokinetic embryonic quality and clinical treatment outcomes were comparable between the three groups.

### Interpretation

In the first study investigating the impact of maternal BMI on morphokinetic parameters, embryos of normal weight and obese infertile donors developed comparably [29]. However, recently a delay in late cleavage divisions (t5, t8) was shown for embryos of overweight and obese women, which is (partially) in contrast to our findings [28]. This study is not directly comparable to ours, as ICSI cycles were studied exclusively, whereas we studied both IVF and ICSI cycles. This may have direct implications, by differences in fertilization techniques, as well as indirect, by differences in study population. In contrast to the study of Bartolacci et al., Leary et al. reported that embryos of overweight and obese women reach the morula stage, and subsequently the blastocyst stage, faster than embryos of normal weight women, although these embryos also have a higher rate of cleavage-stage arrest [35]. Although it is beyond the scope of the current study, the impact of maternal BMI on blastocyst formation rate, an important predictor for implantation, remains inconclusive. Some report no impact or negative impact of high maternal BMI on blastocyst formation, whereas a recent large study reports a higher blastocyst formation rate for obese women [35, 37–41].

It is hypothesized that maternal adiposity may have an effect prior to fertilization. The altered metabolic environment, as a result of an imbalanced diet and chronic excessive oxidative stress, contributes to an abnormal follicular microenvironment [20, 42]. This aberrant microenvironment can derange several pathways including the one-carbon metabolism, which is important for numerous processes involved in reproduction, such as protein and DNA synthesis and redox regulation [43]. This hypothesis is supported by mouse studies showing an effect of obesity on oocyte polarization, reactive oxygen species levels and DNA methylation, including methylation of metabolism-related genes, such as the leptin promotor region [44, 45]. In humans, it has been demonstrated that rising BMI affects regulation of oocyte RNA expression and oocyte metabolism [35, 46, 47]. Furthermore, the maternally-inherited genome passively demethylates with each cell-division, reaching the lowest level at the blastocyst stage, whereas the paternally-inherited genome actively demethylates within 8 h after fertilization [48–50]. Similarly, studies of human preimplantation embryos show that oocyte molecular programs are gradually degraded during the first 3 days after fertilization and those of the embryo genome are activated, culminating between the 4- and 8-cell stage [51–53]. This suggests that early preimplantation embryonic development is primarily driven by the maternal (epi-)genome.

A common cause of female subfertility is polycystic ovarian syndrome (PCOS), which is associated with obesity. PCOS is characterized by a combination of polycystic ovaries, hyperadrogenism and anovulation [54, 55]. Research in women with PCOS undergoing IVF/ICSI shows impaired developmental competence of oocytes, yet preimplantation embryonic development is unaffected [56–59]. In our study, sub-analyses of embryos of women with PCOS demonstrated no significant impact of BMI on morphokinetic parameters (data not shown).

Interestingly, the differences in individual morphokinetic parameters did not translate into differences in the KIDScore distribution. Although the KIDScore is based on only a limited number of parameters, the impact of maternal BMI on these parameters may be too small to induce a shift in the distribution of KIDScores. In embryos of couples with female or combined factor subfertility, however, we found a larger impact of BMI on individual morphokinetic parameters, which may explain the significant negative impact of maternal BMI on the KIDScore. As this is the first study to investigate the impact of maternal BMI on a morphokinetic quality score, comparison to other studies is limited. The KIDScore is a widely applicable morphokinetic (de)selection tool, as it ranks embryo’s according to their implantation potential, regardless of the fertilisation technique used and culture conditions applied [25]. Furthermore, it has a high blastulation predictability and performs superior to conventional morphology evaluation for predicting live births, when applied to day 3 embryos [60]. However, morphokinetic based embryo selection may not be accessible for all fertility clinics, as time-lapse imaging is a relatively expensive technique when compared to conventional culture. In line with this, there are several studies that have investigated the impact of maternal BMI on conventional morphological quality. Although these studies differ in terms of parameters of morphological quality and statistical methods, only one demonstrated an impact of maternal BMI on embryo morphology, suggesting that the impact of maternal BMI on embryonic quality is relatively small [18, 21, 28, 61, 62].

As a tertiary outcome we have addressed the impact of maternal BMI on clinical treatment outcomes in our dataset and found no significant associations. It is very likely that the absence of significant findings can be explained by a lack of power. Yet, the detrimental impact of maternal BMI on success rates of IVF/ICSI treatment has been widely shown in other studies. A recent meta-analysis of over 600,000 women reported a 15% smaller chance of a live birth after IVF/ICSI treatment for obese women compared to normal weight women [63]. Moreover, an additional factor in post-transfer outcomes is the uterine environment. A large retrospective study of over 9,500 normal weight oocyte donors reported lower success rates for obese recipients than for normal weight recipients [64]. Although the exact mechanisms by which obesity alters endometrial receptivity are poorly understood, it is suggested that decidualisation is impaired by genetic dysregulation [65–67].

### Strengths and limitations

By applying a standardized method to measure BMI prior to IVF/ICSI treatment, instead of relying on self-reported data, we reduced the risk of response bias. Moreover, BMI was also categorized according to the World Health Organization classification to facilitate comparison between studies. Statistical strengths are the application of linear mixed model analyses, which takes the clustering-effect of multiple embryos from one women into account, and adjustments for important treatment factors and paternal factors such as age and BMI, so that maternal effects could be studied independently. Another strength is the use of the KIDScore to evaluate embryonic morphokinetic quality at day 3 after fertilization. This deselection tool is universally applicable and has area under the curve of 0.65 for prediction of implantation, which can be considered as a fair predictor [25].

The main limitation of our study is that we have only data until day 3 of development and not until the blastocyst stage, as this is associated with higher rates of pregnancy and live birth [68]. This study was conducted in a time in which fresh transfer of cleavage embryos was routine care in most IVF clinics, including ours, but future research should include embryonic development until day 5. Also, due to the inclusion of IVF treatments, the moment of pronuclear appearance could not be observed in these cases. Although the diverse study population, increases the generalizability of our results, it can also be considered a weakness. As it included both IVF and ICSI treatments and different stimulation protocols, it is a source of possible bias and may elicit divergent results. Furthermore, it is standard care at our clinic to only perform IVF/ICSI treatment in women with a BMI < 34 kg/m^2^, as IVF/ICSI treatment in women with a higher BMI is rarely feasible and associated with increased pregnancy complications [69]. Nonetheless, this practice induces a selection bias for this study. Also, our study population did not comprise any women with underweight. This limited the possibilities to investigate the impact of the full range of maternal BMI. Finally, this study was performed at a tertiary university based hospital. Although not all subfertile couples were in need of tertiary referral or care, our results cannot be automatically extrapolated to the general subfertile population, which may have consequences for the external validity of this study.

## Conclusions

In this study we show that maternal BMI is positively associated with faster progression through the pronuclear and early cleavage stages and negatively with embryo implantation potential. So far, and very likely due to lack of power no associations between maternal BMI and clinical treatment outcomes were observed.

Overweight and obesity are complex diseases and often the result of the interplay between nutrition, lifestyle and genetics. Future research is needed to elucidate the pathophysiological processes involved in the effects of maternal BMI on preimplantation development. Possible explanations might be found in alterations in oocyte quality, DNA damage and decreased cytoplasmic quality, as we observed an impact of maternal BMI on embryo quality in couples with female or combined factor subfertility. In addition, potential metabolic alterations underlying the observed differences in preimplantation development may also have consequences for post-implantation development. Although not demonstrated in this study, the negative effect of increased BMI on ART treatment outcomes has been widely reported. Moreover, maternal overweight and obesity have serious implications for pregnancy outcome and offspring health. Therefore, it is recommended to optimise lifestyle to achieve a healthy weight prior to IVF/ICSI treatment, for example by effective eHealth coaching programs [70, 71]. A healthy weight maximises the general efficiency of the treatment is and minimises alterations in the (early) development of the future generation are minimised.

## Supplementary Information

**Additional file 1: Supplemental table 1.** Morpokinetic parameters of the KIDScore algorithm. **Supplemental table 2.** Female type of subfertility, stratified for maternal BMI.

## Data Availability

The datasets used and/or analysed during the current study are available from the corresponding author on reasonable request.

## Abbreviations

AMH: Anti-Müllerian hormone
BMI: Body mass index
CI: Confidence interval
ET: Embryo transfer
FSH: Follicle stimulating hormone
GnRH: Gonadotrophin-releasing hormone
hCG: Human chorionic gonadotrophin
ICC: Intraclass correlation coefficient
ICSI: Intracytoplasmic sperm injection
IVF: In vitro fertilization
OR: Odds ratio
PCOS: Polycystic ovarian syndrome
Pna: Pronuclear appearance
PNf: Pronuclear fading
