# Supplementary material for: Higher preconceptional maternal body mass index is associated with faster early preimplantation embryonic development: the Rotterdam periconception cohort
Source: Reprod Biol Endocrinol. 2021 Sep 18;19:145. doi: 10.1186/s12958-021-00822-0 (PMC8449446; doi:10.1186/s12958-021-00822-0)
Supplement: Supplementary file 1 — Additional file 1: Supplemental table 1. Morpokinetic parameters of the KIDScore algorithm. Supplemental table 2. Female type of subfertility, stratified for maternal BMI. [file 12958_2021_822_MOESM1_ESM.docx]

**SUPPLEMENTAL MATERIAL**

**Supplemental table 1.** Morpokinetic parameters of the KIDScore algorithm.

| Morphokinetic parameter |
| --- |
| Number pronuclei equals 2 at the 1-cell stage |
| Time from insemination to pronuclear fading (tPNf) |
| Time from insemination to the 2-cell stage |
| Time from insemination to the 3-cell stage |
| Time from insemination to the 5-cell stage |
| Number of cells 66 hours after fertilization |

**Supplemental table 2.** Female type of subfertility, stratified for maternal BMI.

|  | Normal weight  N = 72 | Overweight  N = 39 | Obese  N = 21 |  |
| --- | --- | --- | --- | --- |
|  | N (%) | N (%) | N (%) | *p-*value |
| Uterine factor | 4 (5.6) | 0 (0.0) | 0 (0.0) | 0.179 |
| Tubal factor | 14 (19.4) | 7 (17.9) | 4 (19.0) | 0.982 |
| Endometriosis | 22 (30.6) | 9 (23.1) | 8 (38.1) | 0.459 |
| PCOS | 26 (36.1) | 18 (46.2) | 9 (42.9) | 0.566 |
| WHO-2 non-PCOS^1^ | 9 (12.5) | 3 (7.7) | 0 (0.0) | 0.201 |
| WHO-3^1^ | 2 (2.8) | 1 (2.6) | 1 (4.8) | 0.895 |
| Other^2^ | 0 (0.0) | 2 (5.3) | 0 (0.0) | NA |

The sum of diagnoses is higher than the sum of participants, as some women received multiple diagnoses. PCOS. Polycystic Ovarian Syndrome. WHO, World Health Organisation. ^1^ Stratified according to the WHO classification of anovulation (1). ^2^ Cases included hyperprolactinemia, and ovulation disorder, not specified according to the WHO classification.

1. Dhont M, editor WHO-classification of anovulation: background, evidence and problems. International Congress Series; 2005: Elsevier.
